# Transcranial Motor Evoked Potentials during Spinal Deformity Corrections—Safety, Efficacy, Limitations, and the Role of a Checklist

**DOI:** 10.3389/fsurg.2017.00008

**Published:** 2017-02-13

**Authors:** Shankar Acharya, Nagendra Palukuri, Pravin Gupta, Manish Kohli

**Affiliations:** ^1^Department of Spine Surgery, Sir Ganga Ram Hospital, New Delhi, India; ^2^Department of Anesthesiology, Sir Ganga Ram Hospital, New Delhi, India

**Keywords:** motor evoked potentials, neuromonitoring, spine deformities, transcranial evoked potentials, deformity correction

## Abstract

**Introduction:**

Intraoperative neuromonitoring (IONM) has become a standard of care in spinal deformity surgeries to minimize the incidence of new onset neurological deficit. Stagnara wake up test and ankle clonus test are the oldest techniques described for spinal cord monitoring, but they cannot be solely relied upon as a neuromonitoring modality. Somatosensory evoked potentials monitor only dorsal tracts and give high false positive and negative alerts. Transcranial motor evoked potentials (TcMEPs) monitor the more useful motor pathways. The purpose of our study was to report the safety, efficacy, limitations of TcMEPs in spine deformity surgeries, and the role of a checklist.

**Study design:**

Retrospective review of all spinal deformity surgeries performed with TcMEPs from 2011 to 2015.

**Materials and methods:**

All patients were subjected to IONM by TcMEPs during the spinal deformity surgery. Patients were included in the study only if complete operative reports and neuromonitoring data and postoperative neurological data were available for review. An alert was defined as 80% or more decrement in the motor evoked potential amplitude, or increase in threshold of 100 V or more from baseline. The systemic and surgical causes of IONM alerts and the postoperative neurological status were recorded.

**Results:**

In total, 61 patients underwent surgery for spinal deformities with TcMEPs. The average age was 12.6 years (6–36 years) and male:female ratio was 1:1.3. Diagnoses included idiopathic scoliosis (*n* = 35), congenital scoliosis (*n* = 13), congenital kyphosis (*n* = 7), congenital kyphoscoliosis (*n* = 4), post-infectious kyphosis (*n* = 1), and post-traumatic kyphosis (*n* = 1). The average kyphosis was 72° (45°–101°) and the average scoliosis was 84° (62°–128°). There were in total 33 alerts in 22 patients (36%). The most common causes were hypotension (*n* = 7), drug induced (*n* = 5), deformity correction (*n* = 5), osteotomies (*n* = 3), tachycardia (*n* = 1), screw placement (*n* = 2), and electrodes disconnection (*n* = 1). Reversal of the inciting event cause resulted in complete reversal of the alert in 90% of the times. Three patients showed persistent alerts, out of whom one had a positive wake up test and woke up with neurodeficit, which recovered over few weeks, while the other patients showed persistent alerts but woke up without any deficit. Sensitivity and specificity of TcMEP in deformity correction surgery were 100 and 96.6%, respectively, in our study.

**Conclusion:**

IONM alerts are frequent during spinal deformity surgery. In our study, more than 50% of the alerts were associated with anesthetic management. IONM with TcMEPs is a safe and effective monitoring technique and wake up test still remains a valuable tool in cases of a persistent alert.

## Introduction

Neurological deficit following surgical correction of deformity is a major concern for any spine surgeon (1, 2). Ankle clonus test (3, 4) and Stagnara wake up test (5) are the earliest tests described for assessing the spinal cord function. These tests assess only gross motor deficits and they also require emergence from anesthesia (cannot be applied multiple times) and hence these tests cannot be solely relied upon as a neuromonitoring modality. Role of somatosensory evoked potentials (SSEPs) in spinal cord monitoring was first demonstrated by Tamaki et al. (6) However, there can be a motor deficit without any concomitant sensory change due to vascular injury (7–12). SSEPs have high false positive (FP) and false negative (FN) alerts (12–16) and also need averaging before alerting the surgeon and are thus time consuming (12). Transcranial motor evoked potentials (TcMEPs) on the other hand monitor the more useful motor pathways and are easily administered with high reliability and validity (7). TcMEPs provide feedback almost instantaneously and thus have a good ease of applicability. One of the most important goals of any surgeon performing deformity correction is to maintain the preoperative neurological status (1, 17). Intraoperative neuromonitoring (IONM) system is the means to identify spinal cord injury at the time when corrective measures could reverse it and also to define the nature of insult allowing the surgeon to minimize further injury (18). Controversy still continues regarding the efficacy of TcMEPs alone and few authors prefer multimodality monitoring (19). The purpose of this study was to report the safety, efficacy, and limitations of TcMEPs in spine deformity correction surgeries, and also to establish the role of a checklist.

## Materials and Methods

After approval from Ethics committee (ID: EC/01/17/1107), retrospective review of all spine deformity surgeries performed in our institute during the period 2011–2015 was done. Our study included 67 deformity correction surgeries performed by three senior spine surgeons with a minimum experience of 15 years. Surgeries performed with TcMEP monitoring alone are included in our study. All the surgeries were performed under total intravenous anesthesia (TIVA) protocol developed by the institute, and a trained neurophysiologist who monitors IONM with TcMEPs. Age at the time of surgery, gender, diagnosis, duration of surgery, preoperative neurology, type of instrumentation, blood loss and the number of alerts during surgery, nature of insult, corrective measures done, and postoperative neurology were reviewed. From anesthesia records, depth of anesthesia and mean arterial pressure (MAP) at the time of alert plus anesthesia drug bolus usage were noted.

### Anesthesia Protocol

Before induction, the Stagnara wake up test is explained to each patient; TIVA was employed for induction and maintenance in all the patients. Anesthesia is induced with propofol 1–2 mg/kg i.v., fentanyl 2–3 μg/kg i.v., and dexmedetomidine 1 μg/kg i.v. Intubation is facilitated with only a small, single, short-acting dose of muscle relaxant. The patient’s eyes are taped shut and padded for protection from injury in the prone position. A urinary catheter is placed, an arterial line inserted, two large bore i.v. lines are secured, a temperature probe inserted, and appropriate sized bite blocks are wedged in place between the molars to prevent injury to the contents of the oral cavity (the teeth, tongue, and endotracheal tube). Intraoperative depth of anesthesia was judged by the bispectral index. All used sponges were weighed and saline washes measured, so that accurate assessment of intraoperative blood loss is made. Arterial blood gas analysis and hemoglobin estimations are done as and when required. Anesthesia maintenance is done with i.v. propofol 100–150 μg/kg/h, fentanyl 1–2 μg/kg/h i.v., and dexmedetomidine 0.5 μg/kg/h i.v.

### IONM Technique

Potentials were elicited by transcranial stimulation using corkscrew electrodes placed subcutaneously over the motor cortex (Nim-Eclipse, Medtronic). Motor evoked potentials (MEPs) were obtained from intramuscular electrodes (13 mm, 27G, dual electrodes) placed in four (sometimes five) bilateral muscle groups. One muscle group above the level of surgery was always used as a control (thenar muscles). Other electrodes were placed in rectus abdominis, vastus lateralis, tibialis anterior, and abductor hallucis. The most distal electrodes were placed in the anal sphincter in one case with S2 hemivertebrae. Ultrasound guided placement of electrodes into the rectus abdominis muscle was done in six patients.

Biphasic stimuli were given starting at three pulse, 200 V, and 0.5 ms duration with 2.0 ms interval between stimuli, and if needed increments were done each time by 25 V (up to 400 V) and at five- or seven-pulse train till a satisfactory baseline amplitude (50 μV) was obtained. The same protocol was followed during intraoperative monitoring and the maximal stimulus intensity needed was noted. The initiation of MEP stimulation and recording was done after intubation while the patient was in supine position and once again after patient was placed prone. The MEP recording obtained just before incision was taken as the baseline for future reference. The final MEP was obtained after the closure of wound but before application of dressing.

An “alert” was defined as a decrease in amplitude by 80% or more, or 100 V increase in threshold, or latency prolongation >10% from baseline in one or more electrodes. This need not necessarily be due to a surgical maneuver.

Parallel alert: a similar change (increase/decrease) seen in all the recording electrodes.
Non-parallel alert: a decrease or loss seen in only one or few recording electrodes.

**Figure 1:**
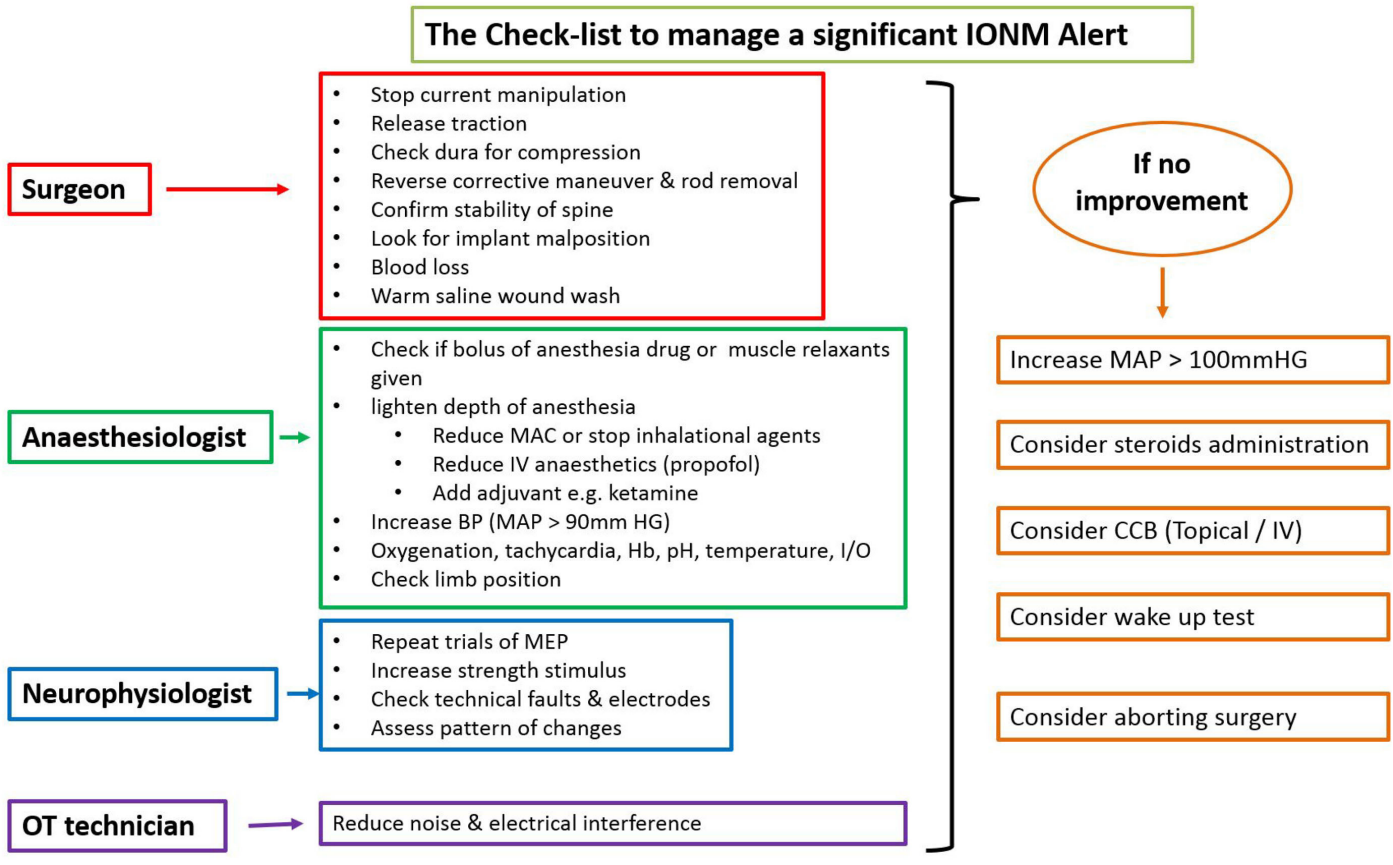
**Checklist showing the protocol taken in response to an alert**.

There is an ongoing protocol in the hospital as a part of neuromonitoring program in Department of Spine Surgery that defined these alerts and also a protocol taken in response to an alert (Figure 1). MEPs were obtained at periodic intervals (10–20 min) during the entire procedure, at lesser intervals during instrumentation, and also immediately after any high-risk maneuver (pedicle breach, distraction, derotation, osteotomy). Once an alert was elicited, the teams performed their set protocols developed by the authors as shown in the checklist (Figure 1).

When an alert was noted immediately following a high-risk maneuver and if recovery of amplitudes was noted after undoing that maneuver, rest of the parameters in the checklist being normal, then it was considered to be the cause. However, if an alert was noted during a routine monitoring protocol, respective teams evaluated all the parameters and the corrective maneuver by which the amplitudes were restored was taken as the most probable cause of an alert.

If the alert persisted even after all the corrective measures were undertaken for up to 30 min, the Stagnara wake up test was done. If the test was negative, the surgery was continued while MEPs were obtained at regular short intervals and if the wake up test was positive, surgery was aborted and the attendants were explained regarding the same.

### Outcome Parameters

The success of IONM (TcMEPs in our study) in determining cord compression at an early stage is expressed with true positive (TP), true negative (TN), false positive (FP), and negative (FN).

TP: an alert that persisted despite corrective measures or returned to baseline after corrective measures, but patient had a positive wake up test (if performed) or postoperative new neurological deficit.
FP: an alert that persisted during surgery despite corrective measures, but patient had a negative wake up test (if performed) or developed no new postoperative deficit.
TN: no alert was recorded during surgery and patient developed no new neurological deficit following surgery.
FN: no alert was recorded during surgery, but patient developed neurological deficit following surgery.
Indeterminate: An alert that returned to baseline value following corrective measures and patient had no new postoperative neurological deficit.

Specificity (Sp), sensitivity (Sn), negative predictive value (NPV), and positive predictive value (PPV) were calculated in our study. Sp and Sn give the percentage of negative and positive outcomes correctly indicated by the technique. PPV and NPV describe the probability that a patient has an injury if the test is positive and does not if the test is negative, respectively. PPV and NPV describe the performance of the technique (chance of a positive or negative neurological event).

Safety was evaluated by observation for scalp burns, arrhythmias, or injuries due to movements induced by TcMEPs like tongue or lip lacerations, seizures, and whether these movements interfered with surgery.

## Results

A total of 67 patients underwent deformity correction surgery with TcMEP monitoring, 6 patients had preoperative neurological deficit and were excluded. A total of 61 patients are included in this study, with an average age of 12.8 years (6–36 years). Most common cause of deformity was idiopathic scoliosis (*n* = 35) (Table 1), other causes being congenital scoliosis (*n* = 13), congenital kyphosis (*n* = 7), congenital kyphoscoliosis (*n* = 4), post-infection kyphosis (*n* = 1), and post-traumatic kyphosis (*n* = 1). Only posterior instrumentation was done in all the cases. Voltage required for obtaining a baseline MEP was generally between 200 and 300 V in our study. Maximal stimulus intensity needed was 200 V in 8 patients (13%), 250 V in 10 patients (17%), 275 V in 16 patients (26%), 300 V in 22 patients (36%), and 350 V in 5 patients (8%) (Table 2). Average kyphosis and scoliosis were 72° (45°–101°) and 84° (62°–128°), respectively.

**Table 1:** **Characteristics of patient population (*n* = 61)**.

Characteristics of patient population	
Age (years)	12.8
Male:female	1:1.3
Diagnosis	No. of patients
Idiopathic scoliosis	35
Congenital scoliosis	13
Congenital kyphosis	7
Congenital kyphoscoliosis	4
Post-infectious kyphosis	1
Post-traumatic kyphosis	1

**Table 2:** **Minimal stimulus intensity required for obtaining baseline potential (*n* = 61)**.

Minimal stimulus intensity required for baseline potential		
	Number (*n* = 61)	Percentage
200 V	8	13
250 V	10	17
275 V	16	26
300 V	22	36
350 V	5	8

**Figure 2:**
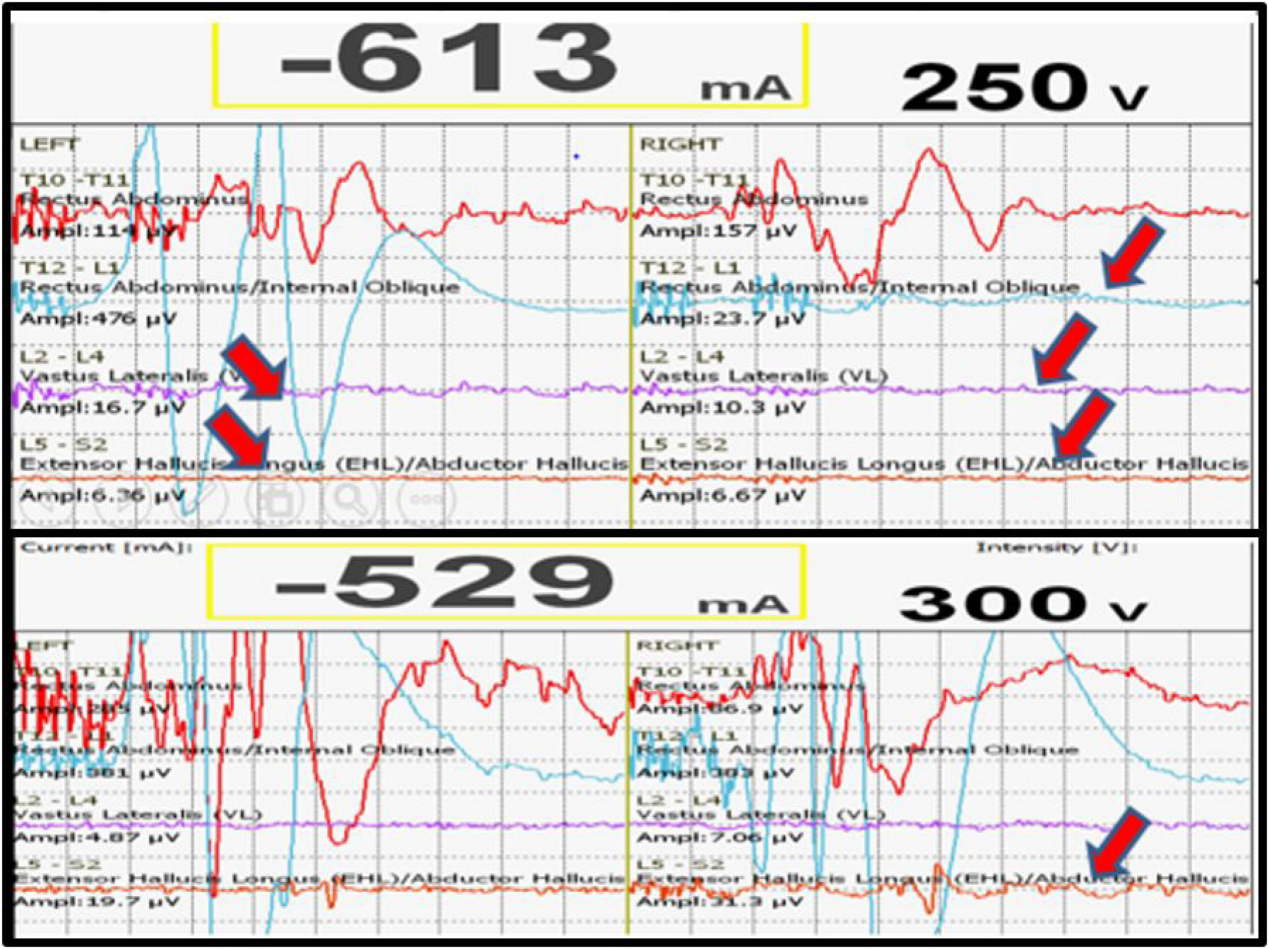
**Loss of MEPs following hypotension (above), recovery of MEPs following correction of hypotension**.

**Figure 3:**
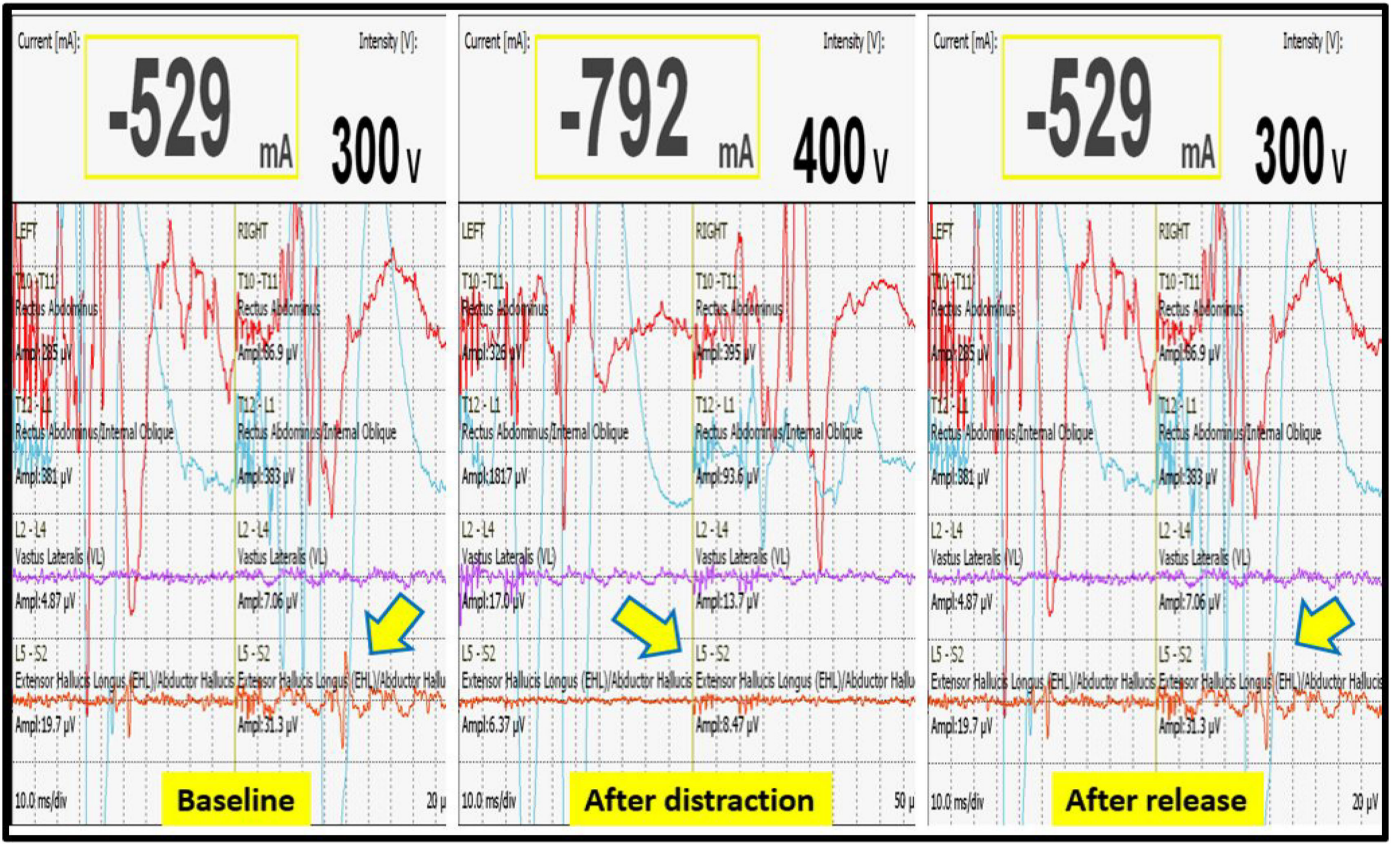
**Decrease in MEP following distraction when compared to baseline value, restoration of MEP after release of distraction**.

**Figure 4:**
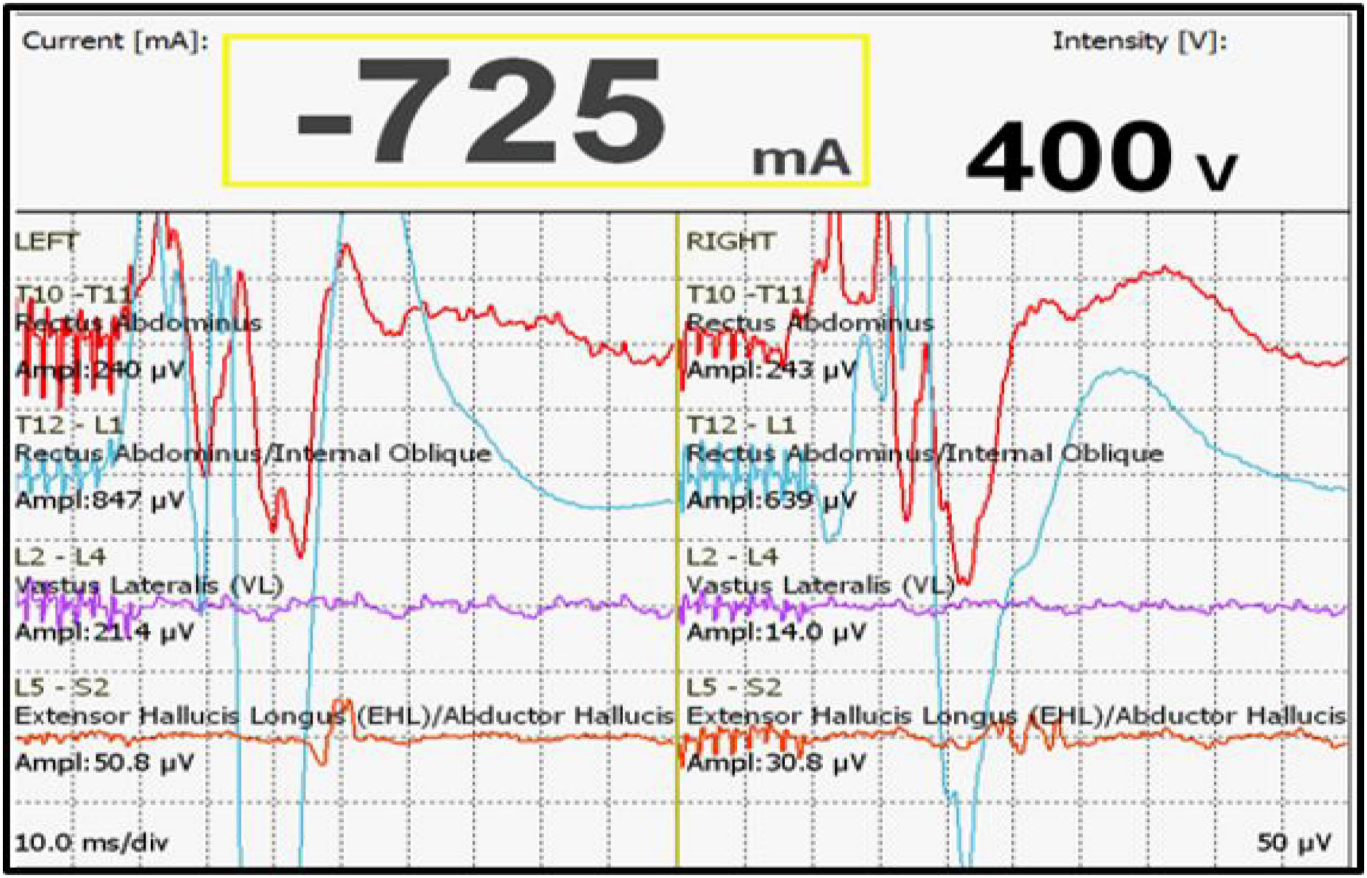
**Loss of MEP due to disconnection of electrodes**.

**Figure 5:**
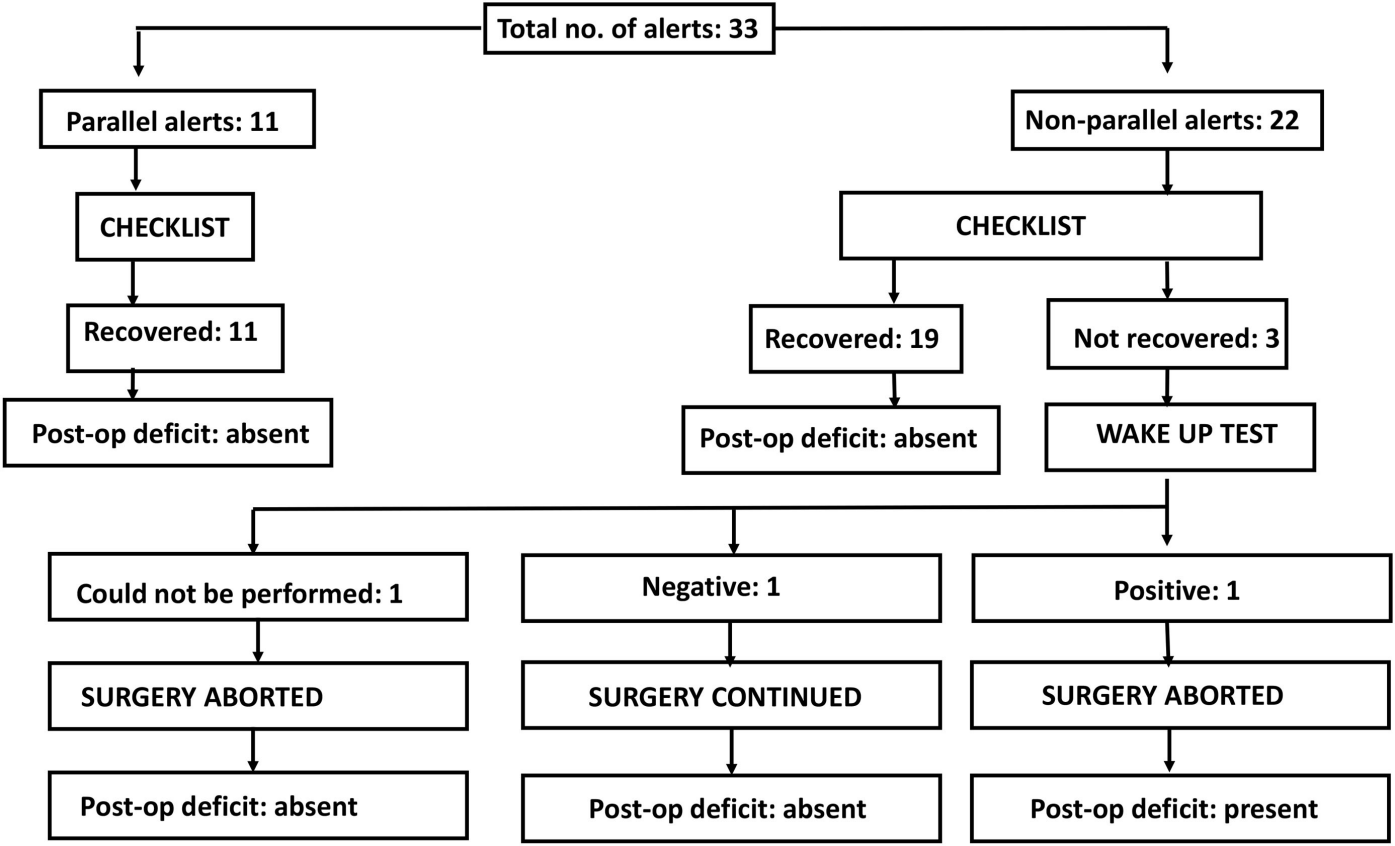
**Flow chart depicting all the alerts**.

We had a total of 33 alerts in 22 patients (36%) (Table 3), 86% (19 out of 22) of alerts returned to baseline values following corrective measures. Eight (25%) alerts were due to altered hemodynamics (hypotension-7, tachycardia-1) (Figure 2). Other causes were anesthetic drug boluses (5, 15%) and distraction of spinal cord (4, 12%) (Figure 3); derotation or deformity correction (5, 15%), osteotomy (3, 9%), hypothermia (1, 3%), screw misplacement (2, 6%), deep stage of anesthesia (2, 6%), and electrode disconnection (1, 3%) (Figure 4). Out of these 33 alerts, 11 were parallel and the rest 22 were non-parallel (Figure 5). We had incidental dural tear in four patients (6.5%), but the authors noted no relation to the alerts.

**Table 3:** **Various inciting events for a TcMEP alert (*n* = 33)**.

Inciting events for TcMEP alert		
	Number (*n* = 33)	Percentage
Hypotension	7	22
Tachycardia	1	3
Drug boluses	5	15
Distraction	4	12
Deformity correction	5	15
Osteotomies	3	9
Screw misplacement	2	6
Deep anesthesia	2	6
Electrodes disconnection	1	3
Hypothermia	1	3
Unknown	2	6

Three patients (13.6%) had persistent alerts; sudden loss of MEPs in both lower limbs was seen in one patient following accidental injury to the spinal cord by pedicle sound through a misplaced screw tract and two patients had decreased MEPs from both lower limbs following distraction. Stagnara wake up test was performed in two patients, out of whom one patient had negative and one had a positive result, while the wake up test could not be performed in one patient. The surgeon decided to continue with surgery in the patient with negative wake up test and MEPs were taken at more frequent intervals; the MEPs restored to baseline value after 50 min and patient woke up with no new neurologic deficit. In the patient with positive wake up test, it was decided to abort the case at that stage and the patient woke up with postoperative deficit. Neurodeficit resolved after a duration of 4 months. Surgery was also aborted in third patient in whom the wake up test couldn’t be done, as the MEPs were persistently low even after all corrective measures had been instituted (Figure 5).

No significant differences were noted in age and gender between patients with no alerts and those who had alerts with or without postoperative deficits. Electrodes were displaced during surgery in three patients, in one case electrodes were reinserted, while in other two cases, surgery was continued without reinsertion. We had no complications with TcMEPs during or after the surgery. All the alerts are shown in Table 4.

**Table 4:** **Complete list of all the 33 alerts in 22 patients**.

S. no. (*n* = 33)	Patient number (*n* = 22)	Age (years)	Diagnosis	Type of alert	Intraoperative motor evoked potential recovery (Y/N)	Cause of alert	Wake up test performed (Y/N)	postoperative Neurological deficit (Y/N)	Recovery at final follow-up
1	1	2–4	Congenital scoliosis	Parallel	Y	Hypotension	N	N	
2				Non-parallel	Y	Osteotomy			
3	2	10–12	Idiopathic scoliosis	Non-parallel	Y	Tachycardia	N	N	
4	3	12–14	Kyphoscoliosis	Non-parallel	**N**	Screw misplacement	**Y**	**Y**	Complete
5	4	4–6	Congenital scoliosis	Non-parallel	Y	Drug bolus	N	N	
6				Non-parallel	Y	Deformity correction			
7	5	50–51	Post traumatic kyphosis	Non-parallel	Y	Distraction	N	N	
8	6	14–16	Idiopathic scoliosis	Non-parallel	Y	Deformity correction	N	N	
9	7	9–11	Idiopathic scoliosis	Parallel	Y	Deep anesthesia	N	N	
10				Parallel	Y	Hypotension			
11	8	13–15	Idiopathic scoliosis	Non-parallel	Y	Screw misplacement	N	N	
12	9	6–8	Congenital scoliosis	Parallel	Y	Drug bolus	N	N	
13				Non-parallel	Y	Hypotension			
14	10	3–5	Congenital kyphosis	Non-parallel	Y	Osteotomy	N	N	
15	11	15–17	Idiopathic scoliosis	Parallel	Y	Hypotension	N	N	
16	12	7–9	Kyphoscoliosis	Parallel	Y	Hypotension	N	N	
17				Non-parallel	Y	Distraction			
18	13	14–16	Idiopathic scoliosis	Non-parallel	Y	Deformity correction	N	N	
19	14	14–16	Idiopathic scoliosis	Non-parallel	Y	Deep anesthesia	N	N	
20	15		Idiopathic scoliosis	Non-parallel	Y	Electrodes misplacement	N	N	
21	16	9–11	Idiopathic scoliosis	Non-parallel	**N**	Osteotomy	**Y**	N	
22				Non-parallel	Y	Drug bolus			
23				Non-parallel	Y	Distraction			
24	17	4–6	Congenital kyphosis	Parallel	Y	Hypothermia	N	N	
25				Non-parallel	Y	Deformity correction			
26	18	10–12	Idiopathic scoliosis	Non-parallel	**N**	Distraction	**–**	N	
27	19	5–7	Congenital scoliosis	Parallel	Y	Hypotension	N	N	
28				Parallel	Y	Drug bolus			
29	20	13–15	Idiopathic scoliosis	Non-parallel	Y	Unknown	N	N	
30				Parallel	Y	Hypotension			
31	21	8–10	Congenital scoliosis	Non-parallel	Y	Deformity correction	N	N	
32				Non-parallel	Y	Unknown			
33	22	6–8	Congenital kyphosis	Parallel	Y	Drug bolus	N	N	

Sensitivity and Sp of TcMEP in deformity correction surgery were 100 and 96.6%, respectively, in our study. NPV and PPV were 100 and 33.3%, respectively (Table 5).

**Table 5:** **Sensitivity (Sn), specificity (Sp), positive predictive value (PPV), and negative predictive value (NPV) of TcMEP alerts**.

	New neurological deficit	No new neurological deficit
TcMEP alert	1 (true positive)	2 (false positive)
No TcMEP alert	0 (false negative)	58 (true negative)
Sn	100%	
Sp	96.6%	
PPV	33.3%	
NPV	100%	

There were no instances of tongue or lip lacerations, seizures, or any other complications during or after the surgery.

## Discussion

The purpose of IONM is to provide real-time assessment of spinal cord function during surgery that involves cord manipulation. Various mechanisms of spinal cord injury in deformity correction surgery are distraction, ischemia, and compression (20). IONM should alert the surgeon of spinal cord injury at a time when corrective measures could reverse it. Patients with congenital scoliosis, kyphosis, and preoperative deficits have a higher chance of neurological injury (1). Stagnara wake up test (5) and postoperative clonus test (3, 4) are the earliest techniques described for knowing the spinal cord integrity during complex deformity corrections. Both tests monitor only gross motor deficits, need emergence from anesthesia and hence they are not real time and cannot be used multiple number of times. There is the risk of self-extubation, loss of patient positioning (21), and is also difficult to perform in some patients (22–24). Despite all these disadvantages, wake up test still has a significant role in certain circumstances; wake up test was done in two patients who had persistent alerts despite corrective measures while wake up test couldn’t be performed in one patient. We routinely do not perform wake up test in all patients, though all except those with low understanding levels, like in very young patients, are counseled for the same. Tamaki et al. in year 1984 first reported the role of SSEPs in deformity corrections (6). SSEPs monitor only dorsal tracts and ventral column can be compromised without a concomitant sensory change (5, 7, 9–12). There are numerous reports of new postoperative deficit in absence of SSEP alerts (12, 16, 25–30). SSEPs require averaging of potentials before an alert is issued and hence lag behind TcMEPs (14, 31, 32). For monitoring with direct spinal cord stimulation (D-wave), electrodes have to be introduced into the dura, and the stimulus unavoidably activates sensory tracts, producing antidromic and peripheral nerve sensory potentials (33). Hence potentials after spinal cord stimulation cannot be attributed to motor tracts alone (34, 35).

In TcMEPs, stimulus is delivered to the motor cortex from subcutaneously placed corkscrew electrodes, and potentials are recorded from electrodes placed in various bilateral muscle groups. The purpose of recording potentials from maximum number of possible muscles is to increase the Sn (36). We used four or five bilateral muscle groups in all cases. Electrodes placed in thenar muscles were always used as a control. In one patient with S2 hemivertebrae excision, the most distal electrodes were placed in anal sphincter on both sides, but during surgery electrodes on one side were displaced, but no attempt was made to reinsert these electrodes. In six patients, ultrasound guided insertion of electrodes was done as rectus abdominis muscle was not easily palpable for direct insertion. Multi-pulse stimulus was used in all cases, as it produces a short train of high frequency stimuli that summate to depolarize motor neurons, thus achieving specific responses (9, 37–42). We used three-, five- or seven-pulse stimulus in all the cases. TcMEPs do not use supramaximal stimuli; hence as depth of anesthesia increases, suppression of lower motor neurons occurs, this may cause disappearance or fading of evoked potentials. Therefore, increasing the pulse number or stimulus intensity may be necessary sometimes to maintain responses (14). In one patient, MEPs were lost from all the electrodes on one side as the patient was at deeper stage of anesthesia. Lack of antidromic contamination in TcMEPs provides Sp and thus is effective and practical in intraoperative period (43). In a direct comparison of TcMEPs with SSEPs, MacDonald and Janusz (44) showed that the former technique provides a rapid feedback.

An 80% or greater decrease in the MEP amplitude to be taken as a criteria for “alert” was introduced by Langeloo et al. (45), while present or absent criteria as an alert was proposed by Sala et al. (46) Various other criteria defined for an “alert” were MEP amplitude changes of 50% (47), 60% (13, 48), 70% (49), and even complete loss (50–53). In our study, alert was defined as a decrease in amplitude by 80% or more, or 100 V increase in threshold, or latency prolongation >10% from the baseline in one or more electrodes.

In all our cases, IONM with TcMEPs was done with strict adherence to anesthesia protocol (TIVA) and checklist. An alert not synchronous with any high-risk surgical maneuver could be likely due to various non-surgical factors and such an alert when not quickly identified and corrected could mislead and compel the surgeon to take unreasonable risk or to change the surgery plan. A checklist places emphasis on all the likely surgical and non-surgical factors that cause an alert and thus a checklist might not allow any potential risk factor to be missed and to mark an alert due to any cause as a FP alert. As the systemic state varies from time to time, baseline potentials obtained at the beginning of surgery may no longer be appropriate at later point. In the intraoperative period, MEP amplitude has high trial-by-trial variability (40) and even a mild drop in MAP can affect MEPs and produce an alert (54–56). These systemic alterations can be identified by parallel alerts, regardless of degree of change in the amplitude. In our study, out of a total 33 alerts, 11 (33%) were parallel and all of these alerts returned to baseline values after restoration of blood volume, increasing the MAP, changing depth of anesthesia, and increasing core temperature. Out of 22 non-parallel alerts seen, 2 (9%) were due to systemic alterations. In our study, all parallel alerts were due to systemic alterations, while non-parallel alerts were due to systemic and focal alterations.

Skinner et al. (57) reported that in some cases, free-running EMGs were the only findings in patients with postoperative neurological deficits. Free-running EMGs were not used in our study, and authors have no experience with these. Relative contraindications to TcMEPs are skull defects, cardiac pacing, epilepsy, and presence of any implantable device (58). Although Schwartz et al. (59) reported no episodes of seizure in 35 patients with a history of epilepsy; TcMEPs were used in only one patient, and epilepsy still remains a contraindication for TcMEPs use in our institute. Wake up test alone cannot be relied as an only monitoring technique as it doesn’t provide real-time assessment of the spinal cord. An alert that persists even after protocol completion could be a FP alert and a negative wake up test can reassure the surgeon of no significant neurological injury (60–62). In three patients who had persistent alerts despite corrective measures, wake up test was performed and out of whom one patient had a negative result. Hence, surgery was continued and the patient woke up with no postoperative deficit. Surgery was abandoned in a patient with positive wake up test, and woke up with deficit in the lower limbs. Wake up test could not be performed in one patient of double major scoliosis due to anesthesia reasons; hence, we decided to abort the case. However, the patient woke up with no new postoperative neurological deficit. Wilson-Holden et al. (63) and Thuet et al. (64) defined alert that normalized after corrective measures and associated with no new postoperative deficit as FP, while Tamkus et al. (65) defined it as TP and Kim et al. (66) as indeterminate. We adopted the definition given by Kim et al. (66) in our study.

We had no FN alerts in our study, although a few case reports exist in literature (52, 67, 68). Sn and Sp of TcMEPs were 100 and 96.6%, while NPV and PPV were 100 and 33.3%, respectively, in our study. According to a meta-analysis by Fehlings et al. (19), many spine centers routinely use multimodality neuromonitoring (TcMEPs and SSEPs) and they also recommend multimodality monitoring in complex deformity surgeries, but FP cases are reported by Hyun et al. even with multimodality neuromonitoring. Absence of any complications or adverse events during or after the surgery, suggest that TcMEPs can be applied safely. In our study, we had high Sn and reasonable Sp with TcMEPs alone; hence, TcMEPs are efficacious for detection of any spinal cord injury in deformity correction surgeries. In patients with neuromuscular scoliosis and in patients with history of epilepsy, we use SSEPs in our institute.

IONM with TcMEPs alone is not without limitations; anesthetic and systemic changes produce high variability in amplitudes, inhalational agents decrease the effectiveness of stimulation, and muscle relaxants inhibit amplitudes from muscles and thus adherence to strict anesthesia protocol is important. Reliability of MEPs diminishes in patients with preoperative neurological deficits. IONM cannot detect abrupt loss of signals as in anterior spinal artery syndrome because this is an acute process. Although TcMEPs have been used without any complications by Schwartz et al. (59) in patients cardiac pace makers, epilepsy, and cardiac disease; they remain contraindications in our institute as patient safety is always paramount. And finally, a FP alert may compel the surgeon to change the surgery plan or to take unreasonable risk. All these limitations have to be kept in mind and also explained to the patient relatives that even in most ideal situations, TcMEPs do not eliminate all adverse neurological events.

### Limitations of Our Study

This study was not a prospective study, study population may not reflect all the deformities (in patients with NM scoliosis and history of epilepsy only SSEPs were used) and no comparison was done with multimodality monitoring. Following an alert, respective teams performed their roles almost simultaneously; hence, the exact cause of an alert may not have been identified all the times. But, as we have an ongoing protocol; with checklist, we think that the point mentioned in the records would most probably represent the cause of an alert.

## Conclusion

In neurologically normal patients, IONM with TcMEPs is a safe and efficacious real-time monitoring system to warn of impending neurological injury at a reversible stage, thus providing a window of opportunity for intervention. Type of alert (parallel or non-parallel) can differentiate systemic and focal compromise. Following the checklist helps in systematically analyzing the potential cause of alert and appropriate action to be taken. Wake up test still remains a valuable monitoring tool in situations of persistent alerts and can help the surgeon in decision-making. Finally, prompt action and close coordination amongst surgeon, anesthetist, neurophysiologist, and operating room staff are required to reduce neurologic mishaps.

## Ethics Statement

This is the standard operating practice; only a standard methodology is assessed in our study.

## Author Contributions

SA – Senior Consultant, Department of Spine Surgery; NP, PG – Spine Fellow, MK – Consultant, Department of Anesthesia.

## Conflict of Interest Statement

The authors declare that the research was conducted in the absence of any commercial or financial relationships that could be construed as a potential conflict of interest.
